# Alleviation of Pain, PAIN Interference, and Oxidative Stress by a Novel Combination of Hemp Oil, Calamari Oil, and Broccoli: A Randomized, Double-Blind, Placebo-Controlled Trial

**DOI:** 10.3390/nu15122654

**Published:** 2023-06-07

**Authors:** Carolina Carlisle, Kristine Polley, Chinmayee Panda, Keri Barron, Meghan Hamrock, Ashley Dominique, Brandon Metzger, Sara Le Brun-Blashka, Slavko Komarnytsky

**Affiliations:** 1 Nutrition Innovation Center, Standard Process Inc., 150 N Research Campus Drive, Kannapolis, NC 28081, USAkbarron@standardprocess.com (K.B.);; 2 Plants for Human Health Institute, North Carolina State University, 600 Laureate Way, Kannapolis, NC 28081, USA; 3 Department of Food, Bioprocessing, and Nutrition Sciences, North Carolina State University, 400 Dan Allen Drive, Raleigh, NC 27695, USA

**Keywords:** whole food, botanical, dietary supplement, bioactive components, antioxidants, hemp oil, pain management, nutrition

## Abstract

Chronic pain is a critical health issue in the US that is routinely managed pharmacologically with diminishing results. The widespread misuse and abuse of prescription opioid pain medications have caused both healthcare providers and patients to seek alternative therapeutic options. Several dietary ingredients have been traditionally used for pain relief and are known to have potential analgesic properties. This double-blind, placebo-controlled randomized clinical trial aimed to test whether a novel combination of full spectrum hemp oil (phytocannabinoids), calamari oil (omega-3 fatty acids), and broccoli (glucosinolates) could reduce chronic pain and attenuate damage from oxidative stress in adults seeking chiropractic care. Participants (average age = 54.8 ± 13.6 years old) were randomly assigned to consume a whole-food, multi-ingredient supplement (*n* = 12, intervention and standard chiropractic care) or placebo (*n* = 13, mineral oil and standard chiropractic care) daily for 12 weeks. The subjects’ self-reported perceived pain, pain interference, and reactive oxygen species (ROS) status in the peripheral blood mononuclear cells (PBMC) were quantified at baseline, mid-checkpoint, and postintervention. The intervention was positively associated with a 52% decrease in pain intensity and several parameters of pain interference, including quality of sleep. Decreases in the markers of oxidative stress were also observed in the participants from the intervention group (29.4% decrease in PMBC ROS). Our findings indicated that supplementation with a novel combination of hemp oil, calamari oil, and broccoli has the potential to manage chronic pain when combined with standard chiropractic care, as suggested by its effects on pain intensity and oxidative stress.

## 1. Introduction

Chronic pain is defined as maladaptive pain that persists for more than 3 months [1]. Over 51 million U.S. Americans (20.9% of its population) suffer from chronic pain, and the Centers for Disease and Control (CDC) estimates that the value of lost productivity due to pain, its direct medical costs, and disabilities program range from USD 560 to USD 635 billion dollars [2]. The impact of chronic pain on a person’s life can be profound, including limitations on daily activities, decreased productivity, increased healthcare utilization, and significant emotional distress, such as depression and anxiety [3]. While pharmacological treatments can be effective in managing pain, they also carry a significant risk of addiction, dependence, and other side effects [4]. The current pain medication crisis, which is exemplified by the widespread abuse of both prescribed opioids and illicit drugs, such as heroin and fentanyl, has a significant impact on public health and healthcare [5]. An increased interest in novel non-pharmacologic and behavioral treatments for chronic pain has been prompted by a national call to action [6], yet effective strategies that can be applied to reduce the use of opioids in chronic pain management are critically lacking.

Chiropractic care is the largest integrative medicine profession in the US, and it attempts to address chronic pain management by promoting the use of non-opioid pain medications, implementing non-pharmacological pain management strategies, and working with patients to develop safe and effective pain management plans [7]. There is increasing evidence that chiropractic interventions are effective in relieving chronic pain [8], especially when combined with lifestyle and nutritional interventions [9,10,11]. The current literature also demonstrates a strong interest from patients regarding the use of opioid-sparing pain management alternatives [12]. Among these, dietary-based interventions that promote successful chronic pain management have become of greater interest [13].

Several dietary ingredients have been traditionally used for pain relief and are known to have potential analgesic properties, including phytocannabinoids from hemp oil [14], omega-3 fatty acids from marine sources such as calamari oil [15], and glucosinolates from cruciferous vegetables, which are most abundant in broccoli [16]. Both phytocannabinoids and omega-3 fatty acids signaling pathways converge in the modulation of the endocannabinoid system (ECS) and the associated inflammatory networks [17]. Inflammation is also closely related to oxidative stress, with inflammatory processes stimulating the generation of reactive oxygen species (ROS) that in turn can trigger further inflammation through the activation of NF-kB and the NLRP3 inflammasome [18]. This concomitantly stimulates the production of the pro-inflammatory cytokines, including tumor necrosis factor (TNF-α), which can increase ROS, resulting in an unresolved cycle of pain, inflammation, and oxidative stress [19,20,21]. Additionally, one of the hydrolysis of glucosinolates is sulforaphane, which, although it has been mainly associated with antioxidant actions, has also been linked to the regulation of inflammatory responses [16].

In this study, we focused on a cohort of subjects with chronic pain enrolled in standard chiropractic care, with or without 12-week nutritional supplementation using a whole-food, multi-component intervention. The primary objective was to quantify changes in the subjects’ self-reported pain and pain interference compared to the study baseline. The secondary objectives were to determine the tolerability of the intervention by assessing adverse event rates and oxidative stress in the peripheral blood mononuclear cells (PBMC).

## 2. Materials and Methods

### 2.1. Study Design and Participants

The study was an adaptive randomized, double-blind, placebo-controlled trial with a parallel assignment, conducted in several North Carolina chiropractic clinics, including the Randolph Chiropractic Center (Charlotte, NC, USA), Trull Chiropractic (Kannapolis, NC, USA), Combined Chiropractic and Acupuncture (Charlotte, NC, USA), and the Nutrition Innovation Center (Kannapolis, NC, USA), from 2018 to 2022. The trial used an adaptive group sequential approach [22], where after analyzing the results from interim analyses, the deletion or addition of treatment arms, and the modification of the dose of the treatment, were considered. Adaptive clinical designs have been successfully used in clinical trials and they are considered an efficient tool for identifying the clinical benefit of the test treatment under investigation [23,24].

**Figure 1:**
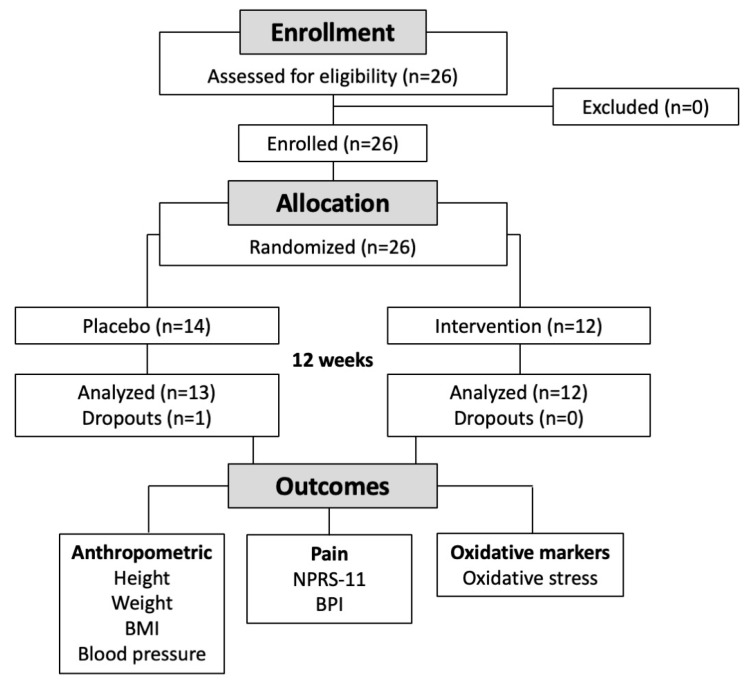
Flowchart of the study.

A flow of the participants throughout the study is shown in Figure 1. Twenty-five potential participants responded to a posting, and all were subsequently offered participation in the study. Participants (18 females, 7 males, age range 37 to 74 years old) were subjects with chronic pain present at two different pain locations. Chronic pain was defined as pain lasting more than 3 months [25]. A combination of two pain locations was selected due to their high relevance to patient lifestyles and the fact that they are difficult to treat [26]. All participants met the inclusion criteria and provided informed consent (Table 1).

At the beginning of the study, subjects were randomized to receive either a multi-component study intervention (study supplement and chiropractic care) or serve as controls (receive matched mineral oil placebo and chiropractic care) daily for 12 weeks. Randomization [27] included the utilization of the 11-Point Numeric Pain Rating Scale (NPRS-11) score upon entry to ensure a similar pain-level between groups and stratification according to the average pain intensity [28,29]. A mid-intervention assessment was also performed at week 6. Throughout the duration of the study, all subjects were instructed to attend a 1-weekly chiropractic session that included a routine procedure of manual chiropractic adjustment, using an activator (a handheld instrument that provides a controlled, low-force adjustment), and arthrostim (a handheld electric instrument that provides a controlled, low-force adjustment). The study was not controlled by diet. Participants were instructed to not make any changes to their dietary intake, lifestyle, or consumption of over-the-counter medication or supplements for the duration of the trial.

All participants completed the study procedures and there were no protocol deviations. The investigators and outcome assessors were blinded to group allocation. The participant information and generated data were fully anonymized for data analysis and the interpretation of results. The study protocol and procedures were reviewed and approved by the Advarra Institutional Review Board (IRB), protocol no Pro00032192, and all clinical investigations were conducted according to the principles expressed in the Declaration of Helsinki. The study was registered on www.clinicaltrials.gov, (Released on 28 March 2023, Identifier: NCT05743855).

### 2.2. Study Investigational Product

The proprietary, whole-food dietary intervention in the form of softgels was supplied by the manufacturer (Palmyra, WI, USA). The serving size was defined as 2 softgels that contained a blend of full-spectrum hemp oil standardized to 15 mg of phytocannabinoids, calamari oil standardized to 230 mg of omega-3 fatty acids, including 130 mg of DHA and 55 mg of EPA, TrueBroc broccoli extract standardized to 5 mg of glucoraphanin, and a carrier oil (extra virgin olive oil). Other ingredients included gelatin, water, dimagnesium malate, glycerin, and beeswax. The subjects were instructed to consume 1 serving per day (2 softgels in the morning or in the evening) for the duration of the study, and to record their daily supplement intake and weekly chiropractic visits in a study calendar to assess their compliance with the intervention.

### 2.3. Anthropometrics

Height, weight, BMI, blood pressure and pulse were measured by the study staff at the beginning, mid-point (week 6), and the end of the study (week 12). Procedures were followed as per the Centers for Disease Control and Prevention guidelines. The Medical Symptoms Questionnaire (MSQ) was administered to all participants at baseline and at the end of the study in order to monitor the subjects’ study readiness, as well as their health-related events during the study.

### 2.4. Oxidative Stress

Blood samples were collected during the study visits in BD Vacutainer CPT mononuclear cell preparation tubes that contained blood separation media comprising a thixotropic polyester gel and a Ficoll Hypaque solution. The tubes were centrifuged to isolate live peripheral blood mononuclear cells (PBMC), and oxidative stress was measured using a fluorogenic cell-permeant probe CellROX Orange (Thermo Fisher, Waltham, MA, USA). Fluorescence upon oxidation by reactive oxygen species was quantified on a BD Accuri C6 flow cytometer using absorption/emission maxima of 545/565 nm and presented as relative fluorescent units (RFUs).

### 2.5. Self-Reported Pain

Subjects were asked to rate their typical pain in the last 24 h on a scale from 0 to 10, where 0 equals “no pain” and 10 is the “worst pain they could imagine”, using the 11-Point Numeric Pain Rating Scale (NPRS-11). NPRS-11 is a validated instrument used to assess pain, and it is considered to show the most sensitivity and stability with regard to chronic pain compared to similar questionnaires [30]. Additionally, the participants were asked to self-report to what extent their pain interfered with different situations in their lives, such as general activity, mood, walking ability, normal work, relationships with others, enjoyment of life, and sleep using the Brief Pain Inventory (BPI) on a scale from 0 to 10, where 0 represented “does not interfere” and 10 represented “completely interferes”. The BPI was originally created in order to evaluate cancer pain and was later validated and used for chronic nonmalignant pain, showing excellent internal consistency for both pain intensity and its interference scale [31].

### 2.6. Statistics

The primary objective of this study was to quantify changes in the subjects’ self-reported pain and pain interference compared to the study baseline, and the secondary objective was to determine the tolerability of the intervention by assessing the adverse event rates and oxidative stress in the peripheral blood mononuclear cells (PBMC). The statistical analyses used the following parameters based on the pilot study data (data on file): the mean of the primary outcome minimum detectable effect size; the standard deviation of the population outcome for the primary outcome; and the levels of significance for the Type I and Type II error rates. They were performed using JMP 15 (SAS Institute, Cary, NC, USA). Power calculations were performed in order to determine that the data analyses would be based on a power of 0.85 so as to detect significant differences between and within the groups using dependent and independent analyses with a level set at *p* < 0.05 (2-sided) [32]. Based on the power calculation, a sample size of 22 was needed (11 for each group), and 1–2 subjects were included in order to account for potential dropouts. All statistical analyses were conducted on an intention-to-treat basis, and values were expressed as means ± standard deviations (SD) [27,33]. Descriptive statistics, as well as two-tailed paired and unpaired student’s t-tests, were used to evaluate changes in the subjects’ clinical outcomes at baseline and after intervention. Statistical significance was set at *p* ≤ 0.05. Asterisks *, **, *** indicate significance levels of *p* < 0.05, *p* < 0.01, and *p* < 0.001, respectively. The blinding was broken only after the data analyses were completed [34].

## 3. Results

### 3.1. Subject Demographics

A total of 26 participants were selected for the study and randomized into the control (*n* = 14, mineral oil placebo) or intervention group (*n* = 12, the study investigational product, a multi-component whole-food intervention that contains calamari oil, hemp oil, and broccoli extract). In total, 13 placebo and 12 intervention group participants completed the study as per the protocol (Figure 1). One placebo subject was dropped from the study due to choosing to continue pain management with a pharmaceutical drug.

The demographics and anthropometrics data of the participants are shown in Table 2. The subjects were well matched between the cohorts based on their baseline demographics and mean pain scores, measured using the numeric pain rating scale (5.8 ± 0.5 versus 5.3 ± 0.6 for placebo) and the brief pain interference scale (5.0 ± 0.5 versus 5.5 ± 1.9 for placebo). There were also no significant differences between groups in terms of pain duration (Table 2).

### 3.2. Tolerance Assessment

No adverse effects attributable to the intervention were reported during the study (no self-reported serious or nonserious adverse events = 0). There were no significant self-reported observations in the compliance calendars and in the Medical Symptoms Questionnaires (MSQ) completed at baseline and at the end of the study, suggesting that all participants followed the study plan provided to them at baseline, and experienced no undesirable events for 12 consecutive weeks of the study. No differences between groups were observed between the groups for the MSQ responses at baseline (*p* = 0.52) and at the end of the study (*p* = 0.27).

### 3.3. Pain Intensity

Participants in both groups presented similar pain locations at entry, with most of the pain reported as back, cervical, neck pain, and muscle and joint pain. For the duration of the study, all participants attended a manual chiropractic adjustment session weekly, independent of the cohort.

**Figure 2:**
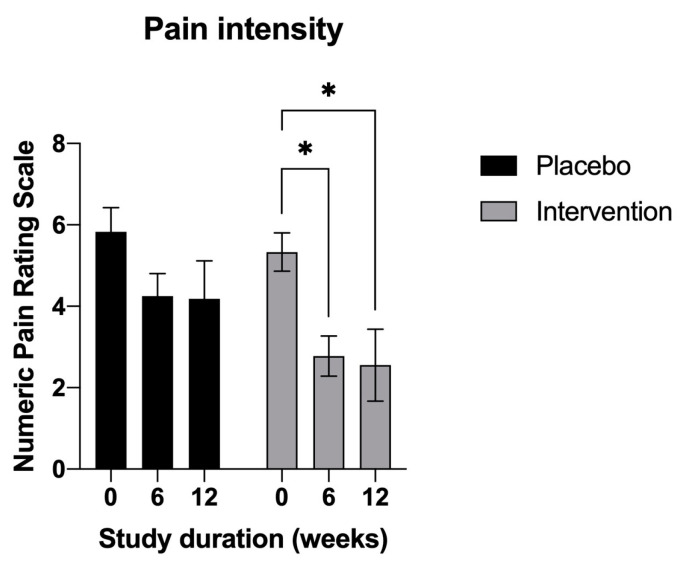
Average numeric pain rating scale scores (NPRS-11) reported at baseline, mid-study (6 weeks) and at the end of the study (12 weeks). Results are expressed as means plus standard deviations. Data were analyzed using two-way ANOVA corrected for multiple comparisons with a Tukey test (* *p* < 0.05).

For the primary outcome measure, the mean plus the standard deviation (±SD) change from the baseline (mean = 5.3 ± 0.6) in the average pain score at 6 (mean = 2.7 ± 1.48) and at 12 weeks (mean = 2.6 ± 2.6) was greatest in the supplemented cohort (−2.5 and −2.7 points, respectively, *p* < 0.05) (Figure 2). The placebo group also experienced a decrease in their pain scores from baseline (mean = 5.8 ± 1.9) to after the 12-week trial (mean = 4.18 ± 3.09), although these were of a lesser magnitude (−1.5 points) and did not reach statistical significance (*p* > 0.05).

### 3.4. Pain Interference

The brief pain interference (BIP) outcome measurements for the baseline, mid-study, and end-of-study checkpoints intended to capture the interference effects of pain on the subjects’ physical health and function, as well as sleep and emotional wellbeing (Table 3).

**Figure 3:**
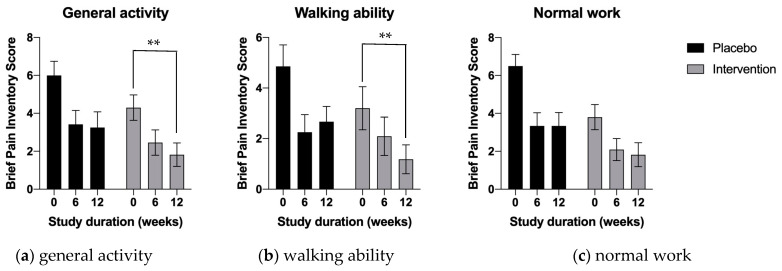
Average numeric brief pain interference scores (BIP) related to physical activity and function at the baseline, mid-study (6 weeks) and end of the study (12 weeks). Results are expressed as means ± SD. Data were analyzed using two-way ANOVA corrected for multiple comparisons with a Tukey test (** *p* < 0.01).

**Figure 4:**
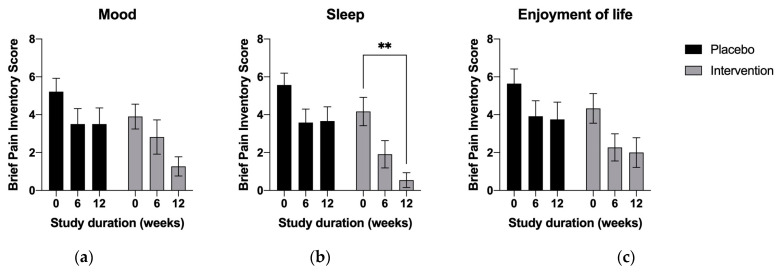
Average numeric brief pain interference scores (BIP) related to sleep and emotional wellbeing at the baseline, mid-study (6 weeks) and end of the study (12 weeks). Results are expressed as means ± SD. Data were analyzed using two-way ANOVA corrected for multiple comparisons with a Tukey test (** *p* < 0.01).

Both at 6 and 12 weeks of supplementation, the measurements tended to demonstrate the benefit of the multi-component calamari oil, hemp oil, and broccoli extract supplementation in all outcome subscales (Figure 3 and Figure 4). This was evident for the reduced effect of pain interference on the subjects’ general activity, walking ability, and normal work; however, they reached statistical significance only for general activity and walking ability (Figure 3a, *p* < 0.01 and Figure 3b, *p* < 0.01 respectively).

The effects were also pronounced in terms of the reduced extent to which pain interfered with the subjects’ mood (Figure 4a), sleep (Figure 4b), and overall quality of life (Figure 4c); however, they reached statistical significance only for mood (Figure 4a, *p* < 0.01) and quality of sleep (Figure 4b, *p* < 0.01). All intervention-related effects tended to increase with time, in contrast to the placebo cohort, which showed no changes between the mid-study and end-of-study checkpoints (Figure 3 and Figure 4).

### 3.5. Biomarkers of Oxidative Stress

**Figure 5:**
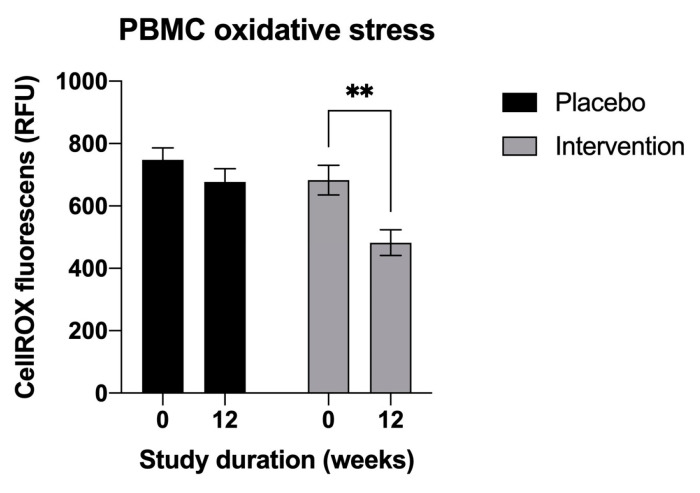
Changes in the oxidative stress biomarker in the peripheral blood mononuclear cells, measured using CellROX Orange reagent. Results are expressed as means ± SD. Data were analyzed using the paired-samples *t*-test (** *p* < 0.01).

At the baseline, the average reactive oxygen species (ROS)-associated oxidative stress in the peripheral blood mononuclear cells isolated from the participants did not differ significantly between the placebo and intervention groups (747.7 RFU and 683.0 RFU, respectively, *p* = 0.26). A 12-week supplementation program was associated with a 29.4% decrease in oxidative stress only in the participants receiving the intervention (482.4 RFU; mean difference −200.5 RFU, *p* < 0.01) (Figure 5). The corresponding ROS reduction in the placebo group was negligible and non-significant (677.2 RFU; mean difference −70 RFU, *p* > 0.05).

## 4. Discussion

While acute pain can provide adaptive benefits to the body, including restricting behaviors that might decrease further damage, chronic pain can lead to long-term behavioral alterations that cause pathological changes in the central nervous system [35]. These changes involve a complex interplay of various physiological and psychological responses that often involve the sensitization of the nervous system (where the pain signaling pathways become amplified and hypersensitive), increased pain perception even in the absence of a noxious stimulus, as well as changes in the inflammatory, oxidative, and neurochemical balances [36]. Individuals may also experience heightened emotional distress, anxiety, and depression, which can further exacerbate the perception and experience of pain. Lifestyle factors such as diet [37], and the utilization of functional doctors, including chiropractors, can affect both the management of pain as well as its underlying or contributing mechanisms [36,38].

Many pharmaceutical interventions that aim to block one of these multifaceted pathways carry significant risks, including the potential for dependence, addiction, tolerance, and overdose, thus limiting their long-term use [39]. The limitations and risks associated with the current medication options highlight the need for alternative approaches that target the complex underlying mechanisms of chronic pain, while minimizing dependence and side effects. Integrative and multidisciplinary approaches, which combine or replace medication with physical therapy, cognitive behavioral therapy, and chiropractic and complementary therapies, are gaining recognition as more comprehensive strategies for managing chronic pain [40].

In the current study, we demonstrated that the use of a whole-food, multi-component nutritional supplement that contained full-spectrum hemp oil standardized to 15 mg of phytocannabinoids, calamari oil standardized to 230 mg of omega-3 fatty acids, and broccoli extract standardized to 5 mg of glucoraphanin decreased the self-reported pain and extent to which pain was said to interfere in the lives of patients living with chronic pain present in two different locations for an average of 14 years. The intervention group demonstrated the greatest change from pain the baseline, both at 6 and 12 weeks of supplementation. Thus, the intervention group experienced, on average, a 52% reduction in self-reported pain intensity at least. This compares favorably with the current IMMPACT recommendation, which considers a 30% decrease in pain severity to be a moderately important and clinically meaningful improvement [41].

Both physical and emotional wellbeing were additionally assessed using the brief pain interference scale as part of a comprehensive evaluation of the intervention outcome. In addition to the perceived pain scores, the greatest significant improvement from the baseline was observed in the subjects’ sleep quality. This finding is further supported by a randomized, placebo-controlled, double-blind study of the hemp oil extract, where overweight, but otherwise healthy, subjects tended to experience an improvement in their sleep [42]. Full-spectrum hemp oils may modulate the autonomic nervous system via endocannabinoid signaling, as there is a well-established role for the ECS in the regulation of pain and sleep responses [17]. One of the mechanisms by which phytocannabinoids may interact with the ECS is by increasing retrograde signaling and reducing excessive neurotransmitter release, thus providing analgesic effects [43]. This effect is directly mediated by the activation of the cannabinoid receptors, which was shown to mitigate neuropathic and inflammatory pain, as well as to be protective against vascular disfunction and ischemia/reperfusion injury [44,45]. It is interesting to note that there is preliminary evidence indicating that chiropractic spinal manipulation therapy may also contribute to pain relief via an increase in endocannabinoids [46], which may partially explain the high number of placebo responders that experienced significant pain relief in the absence of supplementation, as both placebo and intervention groups attended weekly chiropractic adjustment sessions.

Cannabinoid signaling is also intricately connected to omega 6/3 metabolism via the biosynthesis of arachidonic acid and DHA/EPA metabolites that the regulate progression and resolution of inflammation and the associated oxidative stress outcomes [17]. DHA and EPA are metabolized by neutrophils present in the immune system, resulting in interaction with the cannabinoid receptors CB1 and CB2 [16]. This activation, especially of the CB2 receptor on the immune cells, generally results in the inhibition of the inflammatory signaling pathway, such as Tol-like Receptor-4 (CD14/TLR4), which drives the pro-inflammatory immune response by increasing interleukins (IL-1b, IL-6, IL-8) and TNF-α production in macrophages [16]. For this reason, the intervention was also designed to deliver 230 mg of DHA/EPA and 5 mg of glucoraphanin per serving, with the intention of modulating omega-3 metabolism and the nuclear factor erythroid 2–related factor 2 (Nrf2) signaling [16]. Additionally, although best known for being a potent inductor of antioxidant responses, being implicated in the transcription of several antioxidant response genes such as NAD(P) H quinone dehydrogenase 1 (NQO1), heme oxygenase 1 (HO-1), and γ-glutamylcysteine ligase (γ GCL), one of the glucosinolates that hydrolysis produces is sulforaphane, which has also been associated with inflammatory responses [16]. This association is due to its regulation of inflammatory responses via the NF-κB pathway, which is considered one of the classical anti-inflammatory signaling responses [16].

This was evident in part due to the reduced oxidative stress observed in circulating PMBC cells after 12 weeks of supplementation. However, due to the multi-component nature of this intervention, the exact individual effects of its supplementation on the inflammatory and oxidative stress markers may be difficult to predict.

While this study offers novel insights into a whole-food, nutritional intervention, and its potential application as a pain and antioxidant support, it has several strengths and limitations. Chronic pain that has been present for months to years may show an immediate improvement upon the addition of interventions such as chiropractor manipulation, but it is also important that adequate long-term care is provided so that the effects of an intervention can develop and be sustained. However, the conclusions we can draw from this study are limited by the fact that the subject population suffered from chronic pain, a complex and diverse condition, which likely introduced variability into the study.

In summary, our work highlights the feasibility of applying this novel formulation of a whole-food-based, multi-ingredient hemp oil supplementation, and shows that it is capable of supporting the body’s endogenous chronic pain and oxidative networks. The subjects’ response to supplementation was sustained for the duration of the study, showed a tendency to progressively increase, and was also associated with measurable improvements in pain scores, oxidative stress, and sleep quality. These findings were observed in the absence of the apparent adverse side effects. Therefore, this novel nutritional supplementation, based on the combination of hemp oil, calamari oil, and broccoli extract, may benefit subjects who have inadequate chronic pain management or those suffering from the systematic side effects of pain medications.

## Figures and Tables

**Table 1:** Inclusion and exclusion criteria of the study.

Inclusion Criteria	Exclusion Criteria
Male and female individuals aged 25–75 years old Self-reported pain due to a combination of two types of pain related to bone/muscle-related pain and back pain, joint pain and back pain, or cervical pain and joint pain Pain duration of 3 years or more Subjects are not using narcotics to manage pain (anti-hypertensive and lipid-lowering medication allowed) Subjects are not using “warfarin” or any other blood-thinning medication Subjects do not have suspected dementia or other neurological disorders Subjects do not have a pacemaker or any severe medical condition (e.g., insulin-controlled diabetes), or any medical condition that includes serious treatable causes of pain (i.e., cancer, etc.) Subjects must not be pregnant or nursing Subjects must not be a tobacco user Subjects must not have any allergies to fish, hemp	Subjects who are experiencing any adverse events due to any nutraceutical, OTC, pharmaceutical, or investigational products Subjects who self-report a clinically significant condition (e.g., pregnancy, chronic condition, infectious disease, or malignancy, etc.) that, in the opinion of the investigator, would compromise the study or their well-being, or would prevent the participant from meeting or performing study requirements. Subjects that do not comply with the study protocol (i.e., missing study appointments, etc.) Subjects who enroll concomitantly in a different clinical study that, in the opinion of the investigator, would compromise the study or their well-being, or would prevent the participant from meeting or performing study requirements

**Table 2:** Demographic, anthropometric, and baseline data of the study participants (means and standard deviations [±]).

Measures	Placebo (*n* = 13)	Intervention (*n* = 12)
Age (years)	55.1 ± 11.3	55.2 ± 10.7
Gender (female/male, %)	9/4 (69.2%)	9/3 (75.0%)
Hight, cm	165.36 ± 6.54	161.92 ± 4.72
Weight, kg	84.59 ± 20.74	77.57 ± 10.22
BMI, kg/m^2^	31.39 ± 9.76	29.73 ± 4.87
Systolic BP, mmHg	132.72 ± 3.03	125.47 ± 25.50
Diastolic BP, mmHg	91.11 ± 17.01	80.60 ± 11.43
Pain duration (years)	13.9 ± 8.6	14.8 ± 9.5
Pain level (NPRS-11, pts)	5.3 ± 0.6	5.8 ± 0.5
Pain interference (BPI, pts)	5.5 ± 1.9	5.0 ± 0.5
ROS in PMBC	747.7 (±102.1)	682.45 (±106.4)

Abbreviations: NPRS-11—11-Point Numeric Pain Rating Scale; BPI—Brief Pain Inventory.

**Table 3:** Means and standard deviations of the Brief Pain Inventory by group.

BPI	Placebo (*n* = 13) M (SD)			Intervention (*n* = 12) M (SD)		
	Week 0	Week 6	Week 12	Week 0	Week 6	Week 12
General Activity	5.7 (±2.7)	3.4 (±2.5)	3.2 (±2.8)	3.8 (±2.3)	2.5 (±2.2)	1.8 (±2.0)
Mood	5.0 (±2.7)	3.5 (±2.8)	3.5 (±2.9)	3.3 (±2.3)	2.8 (±2.9)	1.3 (±1.6)
Walking Ability	4.7 (±3.2)	2.2 (±2.4)	2.6 (±2.1)	2.7 (±2.7)	2.0 (±2.5	1.9 (±1.8)
Normal Work	6.3 (±2.3)	3.3 (±2.4)	3.3 (±2.4)	3.2 (±2.3)	2.0 (±1.9)	1.9 (±2.0)
Sleep	5.5 (±2.5)	2.5 (±2.6)	3.6 (±2.3)	4.1 (±2.5)	1.9 (±2.3)	0.5 (±2.0)
Enjoyment of Life	5.9 (±2.8)	3.9 (±2.8)	3.8 (±3.1)	4.3 (±2.7)	2.2 (±2.3)	2 (±2.6)

Abbreviations: BPI—Brief Pain Inventory, M—mean, SD—standard deviation.

## Data Availability

Data are contained within the article.

## References

[1] Pizzo P.A., Clark N.M. (2012). Alleviating Suffering 101—Pain Relief in the United States. N. Engl. J. Med..

[2] Dahlhamer J., Lucas J., Zelaya C., Nahin R., Mackey S., DeBar L., Kerns R., Von Korff M., Porter L., Helmick C. (2018). Prevalence of Chronic Pain and High-Impact Chronic Pain Among Adults—United States, 2016. Morb. Mortal. Wkly. Rep..

[3] Volcheck M.M., Graham S.M., Fleming K.C., Mohabbat A.B., Luedtke C.A. (2023). Central Sensitization, Chronic Pain, and Oth-er Symptoms: Better Understanding, Better Management. Cleve. Clin. J. Med..

[4] Nadeau S.E., Wu J.K., Lawhern R.A. (2021). Opioids and Chronic Pain: An Analytic Review of the Clinical Evidence. Front. Pain Res..

[5] Blanco C., Wiley T.R.A., Lloyd J.J., Lopez M.F., Volkow N.D. (2020). America’s Opioid Crisis: The Need for an Integrated Public Health Approach. Transl. Psychiatry.

[6] Darnall B.D., Scheman J., Davin S., Burns J.W., Murphy J.L., Wilson A.C., Kerns R.D., Mackey S.C. (2016). Pain Psychology: A Global Needs Assessment and National Call to Action. Pain Med..

[7] McCubbin T., Kempe K.L., Beck A. (2017). Complementary and Alternative Medicine in an Integrated Health Care Delivery System: Users of Chiropractic, Acupuncture, and Massage Services. Perm. J..

[8] LeFebvre R., Peterson D., Haas M. (2012). Evidence-Based Practice and Chiropractic Care. J. Evid.-Based Complement. Altern. Med..

[9] Coulter I.D., Crawford C., Vernon H., Hurwitz E.L., Khorsan R., Booth M.S., Herman P.M. (2019). Manipulation and Mobiliza-tion for Treating Chronic Nonspecific Neck Pain: A Systematic Review and Meta-Analysis for an Appropriateness Panel. Pain Physician.

[10] Masaracchio M., Kirker K., States R., Hanney W.J., Liu X., Kolber M. (2019). Thoracic Spine Manipulation for the Management of Mechanical Neck Pain: A Systematic Review and Meta-Analysis. PLoS ONE.

[11] Fleming S., Rabago D.P., Mundt M.P., Fleming M.F. (2007). CAM Therapies among Primary Care Patients Using Opioid Thera-py for Chronic Pain. BMC Complement. Altern. Med..

[12] Yang A., Townsend C.B., Ilyas A.M. (2023). Medical Cannabis in Hand Surgery: A Review of the Current Evidence. J. Hand Surg. Am..

[13] De Gregori M., Muscoli C., Schatman M.E., Stallone T., Intelligente F., Rondanelli M., Franceschi F., Arranz L.I., Lorente-Cebrián S., Salamone M. (2016). Combining Pain Therapy with Lifestyle: The Role of Personalized Nutrition and Nutritional Supplements According to the SIMPAR Feed Your Destiny Approach. J. Pain Res..

[14] Odonkor C.A., AlFarra T., Adekoya P., Orhurhu V., Rodríguez T., Sottosanti E., Kaye A.D. (2022). Dorsal Column Stimulation and Cannabinoids in the Treatment of Chronic Nociceptive and Neuropathic Pain: A Review of the Clinical and Pre-Clinical Data. Curr. Pain Headache Rep..

[15] Galán-Arriero I., Serrano-Muñoz D., Gómez-Soriano J., Goicoechea C., Taylor J., Velasco A., Ávila-Martín G. (2017). The Role of Omega-3 and Omega-9 Fatty Acids for the Treatment of Neuropathic Pain after Neurotrauma. Biochim. Biophys. Acta Biomembr..

[16] Santín-Márquez R., Alarcón-Aguilar A., López-Diazguerrero N.E., Chondrogianni N., Königsberg M. (2019). Sulforaphane—Role in Aging and Neurodegeneration. GeroScience.

[17] Komarnytsky S., Rathinasabapathy T., Wagner C., Metzger B., Carlisle C., Panda C., Le Brun-Blashka S., Troup J.P., Varadharaj S. (2021). Endocannabinoid System and Its Regulation by Polyunsaturated Fatty Acids and Full Spectrum Hemp Oils. Int. J. Mol. Sci..

[18] Biswas S.K. (2016). Does the Interdependence between Oxidative Stress and Inflammation Explain the Antioxidant Paradox?. Oxid. Med. Cell. Longev..

[19] Zhang J.-M., An J. (2007). Cytokines, Inflammation, and Pain. Int. Anesthesiol. Clin..

[20] Lim Y.Z., Wang Y., Cicuttini F.M., Hughes H.J., Chou L., Urquhart D.M., Ong P.X., Hussain S.M. (2020). Association Between Inflammatory Biomarkers and Nonspecific Low Back Pain: A Systematic Review. Clin. J. Pain.

[21] Menzel A., Samouda H., Dohet F., Loap S., Ellulu M.S., Bohn T. (2021). Common and Novel Markers for Measuring Inflamma-tion and Oxidative Stress Ex Vivo in Research and Clinical Practice-Which to Use Regarding Disease Outcomes?. Antioxidants.

[22] Bothwell L.E., Avorn J., Khan N.F., Kesselheim A.S. (2018). Adaptive Design Clinical Trials: A Review of the Literature and ClinicalTrials. Gov. BMJ Open.

[23] Cook T., DeMets D.L. (2010). Review of Draft FDA Adaptive Design Guidance. J. Biopharm. Stat..

[24] Chow S.-C., Corey R. (2011). Benefits, Challenges and Obstacles of Adaptive Clinical Trial Designs. Orphanet J. Rare Dis..

[25] Treede R.-D., Rief W., Barke A., Aziz Q., Bennett M.I., Benoliel R., Cohen M., Evers S., Finnerup N.B., First M.B. (2015). A Classification of Chronic Pain for ICD-11. Pain.

[26] Fillingim R.B. (2017). Individual Differences in Pain: Understanding the Mosaic That Makes Pain Personal. Pain.

[27] Dziura J.D., Post L.A., Zhao Q., Fu Z., Peduzzi P. (2013). Strategies for Dealing with Missing Data in Clinical Trials: From De-sign to Analysis. Yale J. Biol. Med..

[28] Broglio K. (2018). Randomization in Clinical Trials: Permuted Blocks and Stratification. JAMA.

[29] McEntegart D.J. (2003). The Pursuit of Balance Using Stratified and Dynamic Randomization Techniques: An Overview. Drug-Form. J..

[30] Lohr K.N., Zebrack B.J. (2009). Using Patient-Reported Outcomes in Clinical Practice: Challenges and Opportunities. Qual. Life Res..

[31] Tan G., Jensen M.P., Thornby J.I., Shanti B.F. (2004). Validation of the Brief Pain Inventory for Chronic Nonmalignant Pain. J. Pain.

[32] van der Meij B.S., Langius J.A.E., Spreeuwenberg M.D., Slootmaker S.M., Paul M.A., Smit E.F., van Leeuwen P.A.M. (2012). Oral Nutritional Supplements Containing N-3 Polyunsaturated Fatty Acids Affect Quality of Life and Functional Status in Lung Cancer Patients during Multimodality Treatment: An RCT. Eur. J. Clin. Nutr..

[33] Armijo-Olivo S., Warren S., Magee D. (2009). Intention to Treat Analysis, Compliance, Drop-Outs and How to Deal with Missing Data in Clinical Research: A Review. Phys. Ther. Rev..

[34] Gariballa S., Forster S. (2007). Dietary Supplementation and Quality of Life of Older Patients: A Randomized, Double-Blind, Pla-cebo-Controlled Trial. J. Am. Geriatr. Soc..

[35] Voscopoulos C., Lema M. (2010). When Does Acute Pain Become Chronic?. Br. J. Anaesth..

[36] van Hecke O., Torrance N., Smith B.H. (2013). Chronic Pain Epidemiology—Where Do Lifestyle Factors Fit In?. Br. J. Pain.

[37] Komarnytsky S., Retchin S., Vong C.I., Lila M.A. (2022). Gains and Losses of Agricultural Food Production: Implications for the Twenty-First Century. Annu. Rev. Food Sci. Technol..

[38] Johnson M.A., Cosgrove C.D. (2015). Complementary and Alternative Medicine for Chronic Musculoskeletal Pain. Fed. Pract..

[39] Bhattacharya D., Whiteside H., Tang E., Kantilal K., Loke Y., Atkins B., Hill C. (2022). A Review of Trial and Real-world Data Applying Elements of a Realist Approach to Identify Behavioural Mechanisms Supporting Practitioners to Taper Opioids. Br. J. Clin. Pharmacol..

[40] Takai Y., Yamamoto-Mitani N., Abe Y., Suzuki M. (2015). Literature Review of Pain Management for People with Chronic Pain. Jpn. J. Nurs. Sci..

[41] Dworkin R.H., Turk D.C., Peirce-Sandner S., Baron R., Bellamy N., Burke L.B., Chappell A., Chartier K., Cleeland C.S., Costello A. (2010). Research Design Considerations for Confirmatory Chronic Pain Clinical Trials: IMMPACT Recommen-dations. Pain.

[42] Lopez H.L., Cesareo K.R., Raub B., Kedia A.W., Sandrock J.E., Kerksick C.M., Ziegenfuss T.N. (2020). Effects of Hemp Extract on Markers of Wellness, Stress Resilience, Recovery and Clinical Biomarkers of Safety in Overweight, But Otherwise Healthy Subjects. J. Diet. Suppl..

[43] Narouze S. (2021). Antinociception Mechanisms of Action of Cannabinoid-Based Medicine: An Overview for Anesthesiologists and Pain Physicians. Reg. Anesth. Pain Med..

[44] Rajesh M., Pan H., Mukhopadhyay P., Bátkai S., Osei-Hyiaman D., Haskó G., Liaudet L., Gao B., Pacher P. (2007). Canna-binoid-2 Receptor Agonist HU-308 Protects against Hepatic Ischemia/Reperfusion Injury by Attenuating Oxidative Stress, Inflammatory Response, and Apoptosis. J. Leukoc. Biol..

[45] Vincent L., Vang D., Nguyen J., Benson B., Lei J., Gupta K. (2016). Cannabinoid Receptor-Specific Mechanisms to Alleviate Pain in Sickle Cell Anemia via Inhibition of Mast Cell Activation and Neurogenic Inflammation. Haematologica.

[46] McPartland J.M., Giuffrida A., King J., Skinner E., Scotter J., Musty R.E. (2005). Cannabimimetic Effects of Osteopathic Manipu-lative Treatment. J. Am. Osteopath. Assoc..

